# Exploring the link between perceived job insecurity and sickness absence for common mental disorders

**DOI:** 10.1093/eurpub/ckaf023

**Published:** 2025-06-10

**Authors:** Sandra Blomqvist, Robin S Högnäs, Kristin Farrants, Emilie Friberg, Linda L Magnusson Hanson

**Affiliations:** Division of Psychobiology and Epidemiology, Department of Psychology, Stockholm University, Stockholm, Sweden; Division of Psychobiology and Epidemiology, Department of Psychology, Stockholm University, Stockholm, Sweden; Division of Insurance Medicine, Department of Clinical Neuroscience, Karolinska Institute, Stockholm, Sweden; Division of Insurance Medicine, Department of Clinical Neuroscience, Karolinska Institute, Stockholm, Sweden; Division of Psychobiology and Epidemiology, Department of Psychology, Stockholm University, Stockholm, Sweden

## Abstract

Perceived job insecurity is associated with poor mental health, but whether it affects sickness absence is not well understood. The present study examines the association between perceived job insecurity and sickness absence due to common mental disorders and whether changes in perceived job insecurity affects the risk of sickness absence due to common mental disorders. Data are from the Swedish Longitudinal Occupational Survey of Health and include those who participated at least once between 2010 and 2020 (*n* = 24 049). Two different types of analyses were conducted: (1) logistic regression with adjustments for baseline covariates and (2) pooled logistic regression with inverse probability weights, across 5 emulated target trials assessing onsets and/or offsets of job insecurity versus stable security or stable insecurity, on the risk of sickness absence for common mental disorders. Perceived job insecurity was associated with increased odds of sickness absence for common mental disorders over a 2-year period (odds ratio = 1.38, 95% confidence intervals (CI) 1.13-1.68). We found no statistically significant associations for an onset of job insecurity versus being stably secure (risk ratio (RR) 1.484, 95% CI 0.913-2.055) nor for offset versus stable insecurity (RR 0.855, 95% CI 0.308-1.402). The findings from our emulated target trials were, however, uncertain. Findings suggest that perceived job insecurity increases the risk of sickness absence for common mental disorders. The study implies that efforts to increase employee’s sense of security may help reduce rates of sickness absence for common mental disorders if job insecurity is reduced long-term.

## Introduction

Mental health problems account for a substantial share of the total burden of disease [1] and can negatively affect one’s quality of life, work ability, personal finances, and relationships [2]. It is also costly for society as common mental disorders (CMD), e.g. depressive, anxiety, and stress-related disorders, often lead to sickness absence (SA) and/or disability pension (DP). In 2015, the associated economic burden exceeded 4% of Sweden’s Gross Domestic Product, similar to the EU average, of which a large share was due to illness-related financial insecurity [3].

At the same time, work is viewed as a means of reducing the growing old-age dependency ratio, and the rising costs of pensions and the healthcare system. A balance between employers’ flexibility and employee security is believed to improve labor market participation and economic growth [4]. An imbalance, however, may increase both objective and subjective job insecurity, and with negative mental health consequences. Research shows that job insecurity is associated with depression [5], anxiety [6], purchases of psychotropic drugs [7, 8], suicide ideation [9], and suicide death [10].

Other working conditions such as high job strain, effort-reward imbalance, bullying and low support have been consistently associated with SA [11, 12]. Estimates suggest that 30% of SA could be prevented by improving psychosocial work environments [13]. However, only a few studies have examined job insecurity and SA, with mixed findings—some found that job insecurity increased the risk of SA [14, 15], another found no association [16], and others found job insecurity to increase sickness presenteeism [11, 17]. Moreover, women with high job insecurity versus job security had a lower risk of SA, while the opposite was the case for men [11]. These sex differences, however, have not been replicated in other studies [15, 16].

More research is warranted given the salience of these issues for individuals and societies. Specifying the diagnosis for an SA spell, can help elucidate the relationship between job insecurity and mental health in general and for sickness absence in particular. To our knowledge, only one study has examined job insecurity and SA due to mental disorders and found no association among male manual workers in Japan [18]. Therefore, this study extends the literature by using panel data from Swedish employees to assess the role of job insecurity in SA due to CMD, including the impact of changes in job insecurity over time.

## Methods

### Data

Data are from the Swedish Longitudinal Occupational Survey of Health (SLOSH) between 2010 and 2020, *n* = 28 808 [19]. SLOSH consists of an originally nationally representative sample drawn from the Swedish Work Environment Survey. Participants have been asked to respond to SLOSH questionnaires biennially, with response rates between 43% and 65%. SLOSH data are linked to several national Swedish registers. We used data from the Micro-Data for Analysis of the Social Insurance System (MiDAS) from the National Social Insurance Agency. We included those with at least one wave of information on job insecurity (*n* = 24 049), and (due to rules for SA) ≤63 years old (*n* = 22 394), not self-employed nor partly retired and without SA due to CMD 2 years prior to measuring job insecurity. The analytic sample included *n* = 20 882 individuals and 54 500 person-observations (Supplementary Fig. S1).

### Perceived job insecurity

SLOSH respondents were asked how much they agreed with: (1) feeling worried about being dismissed, (2) worrying about getting to keep their job, and (3) feeling afraid of losing their job (from 1 = not at all to 5 = fully). Scores ≥4 on ≥1 item were classified as job insecurity. Scores ≤3 on all items were classified as job security. These items have been validated, yielding satisfactory factor loadings, internal consistency, and explained variance [20].

*Job insecurity onset* was defined as no job insecurity in the first of 2 consecutive waves (*T_x_*), but a perception of job insecurity in the subsequent wave (*T_x_*_+1_). The reference group included those unexposed to job insecurity across both waves (i.e. “stably secure”). Conversely, *job insecurity offset* was defined as exposure to job insecurity at *T_x_* and unexposed at *T_x_*_+1_ (ref. = stably insecure).

### Sickness absence due to common mental disorders

SA due to CMD was measured using start and end dates and the main diagnosis and obtained from MiDAS for spells ≥14 days. Only spells with ICD-10 diagnosis codes F30-48 were included, covering, e.g. affective disorders, stress-related, and other nonpsychotic mental disorders, where 1 = SA due to CMD between the date of job insecurity and the subsequent SLOSH wave and 0 = no SA due to CMD. We censored individuals at death, DP, or end of follow-up.

### Potential confounders

Sex, age, education, occupational class, country of birth (Sweden/outside of Sweden), employment sector, type of living area, and civil status (married or cohabiting/other) were measured at baseline. We excluded employment contract (fixed or permanent) due to multicollinearity issues with education, occupational class, sex, and age. Moreover, contract type was uncorrelated with the outcome and thus was not considered as a confounder [21].

### Statistical analysis

First, we assessed perceived job insecurity and the risk of SA due to CMD using generalized estimating equations (GEE), which allow for adjustments for multiple observations per person by applying a working correlation structure. We compared quasi-likelihood under the independence model criterion (QIC), which is similar to Akaike’s information criterion (AIC), but it is based on quasi-likelihood assumptions. We evaluated different options and selected the independent type as the correlation structure [22]. We compared the crude and fully adjusted models, stratified by sex and included an interaction term to examine sex as an effect modifier of the relationship between job insecurity and SA.

Second, random and fixed effects models were estimated to assess between- and within-individual variation, respectively. As the FE models exclude cases with no change in the exposure, the statistical power was substantially reduced, which made the results difficult to interpret in a meaningful way. Therefore, we employed an alternative analytic approach. Target trial emulations (TTE) estimates the effect of change in the exposure on potential changes in the outcome, but compares individuals whose perceived job insecurity changed to those whose perceptions did not change [23]. This effectively simulates what would happen, hypothetically, if we intervened on perceived job insecurity while retaining more observations in the analysis.

To assess the impact of job insecurity changes across 2 years (ref. = stable security or stably insecure) on the risk of SA study protocol in Supplementary Table S1), we composed 5 trials between 2010 and 2020. Survival time was observed from “treatment” assignment until SA or censoring (Supplementary Fig. S2 and Table S2). If the inclusion criteria were met, a person could be included in multiple trials, which improves statistical efficiency. Robust standard errors to calculate 95% confidence intervals (CI) were obtained using 500 iterations of bootstrapping [24–26]. The trials were pooled and individuals received one row per month of follow-up starting at *T_x_*_+1_ until the first month of SA due to CMD, censoring, or end of follow-up (≈730 days). This was repeated over each trial for a given person. Pooled logistic regression was estimated with intention-to-treat effects on the risk of SA due to CMD at 12 and 24 months [27]. The assumption of rare events was met within the monthly intervals, and thus, odds ratios approximated relative risks (RR) [26], which are reported. Standardized survival curves show the risk for SA over time by exposure groups (i.e. onset, offset, stably secure, and stably insecure) [26].

Exchangeability between exposed and unexposed groups was strengthened using inverse probability weighting (IPW) based on baseline covariates [27]. IPW yields a pseudo population in which baseline covariates are independent of exposure levels [27], which was used to calculate the average counterfactual survival probabilities by exposure level and the risk ratios [25]. As robustness checks, we relaxed the inclusion criteria for no SA due to CMD prior to the first (*t_x_*) of the 2 measurement points, which were required to define treatment assignment and start of follow-up (*t_x_*_+1_). We censored individuals at SA for diagnoses other than CMD and increased the contrast between the treated and the untreated groups by dropping neutral responses (i.e. a “3”) on all three 5-point Likert scale items. Non-exposure to job insecurity was set to ≤2 on all items and exposure to ≥4 on at least one item. Analyses were conducted using Stata 16, partly based on previous literature [25, 26].

## Results

Sample characteristics by exposure are presented in Table 1. In total, *n* = 20 882 unique individuals contributed to 54 500 observations, of which 6% perceived job insecurity. The most striking difference was the larger proportion of private sector workers among those with, versus those without, perceived job insecurity (Table 1). Overall, there were more women than men (58%) in the sample, but the proportion of women was slightly smaller in the job insecurity versus the secure group. The job insecurity group generally had lower education, more manual workers, individuals who were younger, born abroad, and who lived without a partner. The prevalence of SA due to CMD was 4% among those with job insecurity, compared to 3% among those without job insecurity.

**Table 1.** Person observation characteristics among individuals participating ≥1 wave in SLOSH with information about job insecurity

	Job insecurity		No job insecurity	
	No. Obs	%	No. Obs	%
	3298	6	51 202	94
Number of unique individuals	1581		19 301	
Sickness absence for common mental disorder within 2 years after job insecurity assessment	140	4	1713	3
Women	1836	56	29 597	58
Age	49	9	50	9
Married/cohabiting	2490	76	41 179	80
Born in Sweden	2994	91	47 999	94
*Residence*				
Large city or nearby municipality	1158	35	15 901	31
Medium-sized town or nearby municipality	1217	37	20 941	41
Small town or nearby municipality	923	28	14 360	28
*Highest level of education*				
Compulsory	1102	33	14 970	29
Upper secondary	721	22	8480	17
University	1471	45	27 724	54
*Type of occupation*				
Manual employee	1112	35	14 303	29
Non-manual employee	2107	65	35 812	71
*Sector of employment*				
Private	1714	64	21 723	52
Public	975	36	20 202	48
Step 2 analysis				
*Job insecurity status across 2 consecutive waves*				
Onset	994	30	na	
Stably secure	na		25 975	51
Offset	1207	37	na	
Stably insecure	426	13	na	

### Logistic regression analyses

Apart from the FE model, findings were consistent in all models, suggesting that job insecurity increases the subsequent 2-year risk of experiencing SA due to CMD (Table 2). After adjusting for time stable and time-varying factors, RE and GEE estimates were OR 1.38, 95%CI 1.13-1.68. We did not find effect modification by sex; thus, these results are not shown. The FE results suggest no association between job insecurity and SA due to CMD (OR 0.83, 95%CI 0.59-1.17), but the number of observations was also considerably reduced in this model.

**Table 2.** Estimates with logistic regression models on prospective cohort data

	Unadjusted GEE model		Fully adjusted^a^ GEE model		Random effects^a^		Fixed effects^b^	
Person observations	54 500		43 816		43 816		2797	
No. clusters (individuals)	20 882		19 507		19 507		1 002	
	OR	95%CI	OR	95%CI	OR	95%CI	OR	95%CI
No job insecurity	ref.		ref.		ref.		ref.	
Job insecurity	1.28	1.07-1.53	1.38	1.13-1.68	1.37	1.10-1.69	0.83	0.59-1.17

a Adjusted for sex, age, region of living, cohabitation, country of birth, education, occupational class, and sector of employment.

b Adjusted for region of living, cohabitation, education, occupational class, and sector of employment.

### Target trial emulation

The distribution of sociodemographic factors was similar in the analytic samples assessing onset and offset of job insecurity. Individuals who experienced an onset (person-observations = 994) or who remained stably insecure (obs. = 426) across 2 years were largely men, private sector workers, individuals with lower education, manual workers, and those living without a partner, compared to the offsets (obs. = 1207) and the stably secure (obs. = 25 975). There were no major differences in prevalence of SA due to CMD (Supplementary Tables S3 and S4).

**Figure 1.**
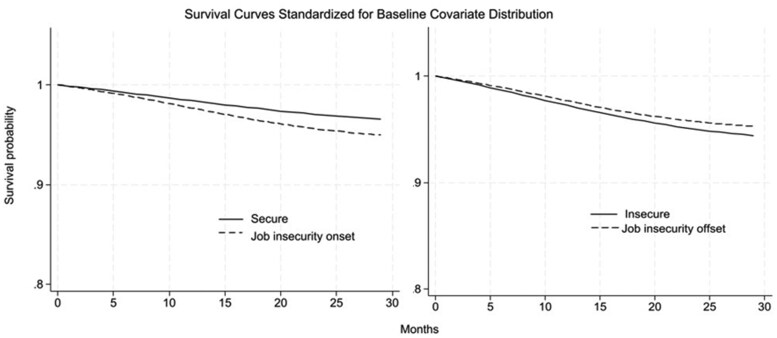
Survival curves for sickness absence due to common mental disorders, by level of treatment. Solid lines represent the stable groups and the dashed those with a job insecurity change.

Figure 1 shows survival curves for SA due to CMD, standardized by baseline covariates. Towards the end of the follow-up, job insecurity onsets had a lower probability of “surviving”, i.e. remaining free of an SA spell due to CMD, than those who were stably secure across 2 waves. Conversely, the probability of surviving seemed higher when job insecurity was removed versus when it persisted. However, confidence intervals around the survival probabilities overlapped, and thus, no statistically significant distinctions were supported (Table 3).

**Table 3.** Target trial emulation for population average intention-to-treat effects

	After 12 months of follow-up			After 24 months of follow-up		
	RR (95%CI)	Counterfactual HR (95%CI)	Survival probability (95%CI)	RR (95%CI)	Counterfactual HR (95%CI)	Survival probability (95%CI)
*From secure to insecure*						
Person obs = 26 969						
Unique individuals = 12 129						
Stably secure	Ref.	Ref.	0.984 (0.981-0.987)	Ref.	Ref.	0.970 (0.967-0.972)
Onset of job insecurity	1.468 (0.897-2.039)	1.474 (0.881-2.066)	0.977 (0.958-0.996)	1.484 (0.913-2.055)	1.495 (0.903-2.088)	0.955 (0.936-0.974)
*From insecure to secure*						
Person obs = 1633						
Unique individuals = 1347						
Stably insecure	na	na	na	Ref.	Ref.	0.950 (0.922-0.978)
Offset of job insecurity	na	na	na	0.855 (0.308-1.402)	0.852 (0.291-1.412)	0.957 (0.943-0.971)

Pooled logistic regression models on person-time data comparing changes in job insecurity status (onset and offset) to stability (stably secure and stably insecure) across 2 years and subsequent risk for sickness absence due to common mental disorders. Risk ratios and counterfactual hazard ratios, adjusted for baseline confounders by inverse probability weighting, truncated at the 99th percentile are presented with robust 95% confidence intervals from 500 bootstrap samplings.

We found no statistically significant intention-to-treat effects for job insecurity onsets compared to remaining secure on the risk of experiencing SA due to CMD after 24 months of follow-up (RR 1.484, 95%CI (0.913-2.055), counterfactual HR 1.495 (0.903-2.088). There also was no statistically significant association between offsets of job insecurity versus stably insecure and the risk of SA due to CMD (RR 0.855 95% CI 0.308-1.402). However, these estimates were highly uncertain, particularly for offset of job insecurity (Table 3).

Overall, results from the sensitivity analysis were consistent with the main analysis. The precision improved slightly when relaxing the criteria for prior SA due to CMD before baseline as more cases were retained, but the point estimates were also smaller compared to the main analyses (Supplementary Table S5).

The number of observations for offset was limited, so we restricted additional tests to onset of job insecurity. Censoring individuals at a non-CMD diagnosis SA event reduced RR to 1.39 (95%CI 0.78-2.01). However, increasing the contrasts between job insecurity onset and the stably secure, resulted in similar estimates as the main analysis although with a further loss of precision (RR 1.49 95% CI 0.75-2.22; Supplementary Table S6).

## Discussion

This study found an excess risk of SA due CMD over a 2-year period among employees who experienced job insecurity compared to those who experienced security. However, neither onsets nor removal of job insecurity over 2 years, compared to remaining stably secure or insecure, respectively, was significantly associated with changes in SA due to CMD. Likewise, once we adjusted for fixed unobserved characteristics in our fixed effect models, the association we observed in the logit and random effects models, which do not account for unobserved heterogeneity, was no longer statistically significant. This could indicate that it is not the change per se, but rather the time spent as insecure, that increases the risk of SA due to CMD. This is consistent with studies showing that chronic job insecurity has a stronger negative impact on mental health than a single time exposure [28]. This also resonates with what our outcome represents—sickness absence >14 days more likely captures persistent mental health problems that have become difficult to cope with alongside other obligations (e.g. work), than more transient problems. However, this may also be due to the reduced sample size in the TTE and FE analyses, which makes a causal interpretation of our findings difficult. While the adjusted FE and TTE estimates are not statistically significant, the RRs are greater than one (e.g. RR 1.48 in the TEE model), suggesting that they may still be substantively meaningful. It is not possible to discern whether the level of uncertainty around the estimates is due to the sample size, residual sources of bias such as selection or confounding, or whether job insecurity has no causal effect on SA due to CMD.

The direction of point estimates, however, suggests that job insecurity indeed increases the risk of SA due to CMD and corroborates prior findings that job insecurity is associated with symptoms of depression [5], anxiety [6], psychotropic drug purchases [7, 8], suicide deaths [10] and sickness absence in general [14, 15]. In contrast to one previous study [11], we found no sex differences with regard to job insecurity and risk of SA due to CMD. Furthermore, the effect size and level of uncertainty in the current study were similar to those in a previous study showing that moderate job insecurity increased SA due to mental disorders [18], although our job insecurity measurements were different. Our measurement captures affective job insecurity [29], which assesses an individual’s fear of a job loss, whereas Inoue *et al.* [18] measured the perceived likelihood of employment discontinuity (i.e. cognitive job insecurity) [29]. When compared, affective job insecurity is generally more strongly associated with adverse mental health outcomes than cognitive job insecurity [30].

Inconsistent with the Japanese study [18], we did not find that higher levels of job insecurity decreased risk of sickness absence for depressive symptoms. It is noted elsewhere [14] that job insecurity has both a disciplining and stressor effect on sickness absence, which is certainly possible here. Also, in the presence of other conditions, like high financial insecurity or low employability, sickness absence behaviors may be different [31, 32] or vary across different institutional settings [33].

SA due to CMD is common and costly for individuals, organizations, and societies. Therefore, the current findings need to be assessed in relation to efforts to improve perceived job insecurity, and the consequences of not doing so. Studies show that perceived job insecurity can be targeted through interventions at the organizational level amid downsizings [31, 34], and on a national level through generous social safety nets [35]. The latter would preferably be combined with policies promoting employment mobility [36] and training to increase employability [37]. These policies potentially have indirect positive effects on health and well-being, and by extension sickness absence, which was the case for the unemployed who participated in previous active labor market programs [38].

### Strengths and limitations

With access to dates on medically certified SA spells from a population register in Sweden, there was virtually no follow-up loss on the outcome variable, except for those who emigrated. Coupled with survey data on exposure to job insecurity, the risk of common method bias was reduced. However, due to SLOSH attrition, there may still be sample issues that produce selection bias. By restricting our analysis to cases of SA due to CMD according to the ICD-10 classification system of diseases, we contribute to a better understanding of the link between job insecurity and SA. This is an advancement of the knowledge in this field, as prior studies have focused on the risk of SA from all diagnoses, jointly. We relied on multiple items to measure job insecurity, which, compared to using a single item, is less susceptible to random measurement errors. Both multiple item constructs [39] of job insecurity and affective job insecurity [30] are generally more strongly associated with mental health outcomes, compared to single items or measures of cognitive job insecurity. Thus, both the conceptualization and operationalization employed in this study are a more robust approach to assessing the impact of job insecurity on SA due to CMD.

In the sensitivity analysis, individuals with SA due to non-CMDs, most often musculoskeletal, were censored because they were no longer at risk for a CMD given the structure of the register. This decreased statistical power and potentially the effect size. In several of the cases that were lost, a relatively short time elapsed between the different spells, hence the independence of these spells was then questionable. Thus, the temporally close spells may have been susceptible to diagnosis misclassification, while our individual-level measure of perceived job insecurity, compared to exposure matrices from prior studies [11], likely reduced the risk of misclassification.

The logistic regression models include exposed and unexposed individuals with highly varying lengths in their exposure status. Some of the exposed may have experienced job insecurity for decades, while others only transiently, but experience job insecurity that coincides with SLOSH data collection. Correspondingly, some of the unexposed may never perceive their jobs as insecure, while for others, the data collection time point may be atypical for their generally insecure job situation. Therefore, our examination of the changes in job insecurity status and sickness absence is an important complement, which strengthens the study by creating better control over the type of exposure levels that are compared. Causal interpretations are facilitated by investigating the impact of a change in one factor in *one population*, on the change in another subsequent factor in that same population [26]. Following a change reduces the risk of selection bias that may otherwise arise when categorizing individuals as exposed or unexposed based solely on their baseline status—a bias which can be further exacerbated when imposing additional inclusion criteria, such as having no SA due to CMD.

Alignment between study eligibility, exposure assignment and start of follow-up (time zero) is a key in TTEs [23]. Unfortunately, this could not be entirely done in the present study due to the nature of the SLOSH data collection. Job insecurity status was measured every 2 years, and we do not know the exact timing of the perceived change, and thus, the length of the time gap to the start of follow-up of SA. Another potential source of bias is immortal time bias [23], as individuals with SA due to CMD during 2 years prior to, or during, the period of treatment assignment and start of our follow-up were excluded from the analysis. When this was relaxed by only excluding those who had SA due to CMD during the 2 years used to determine job insecurity onset and offset, the effect size attenuated. However, the RRs were in the same direction and with slightly narrower confidence intervals given the larger sample size.

The TTE design was employed to strengthen our ability to make causal inferences and to reduce some of the systematic biases inherent to observational studies. However, the present study may still be susceptible to unobserved confounding, making it difficult to establish causation. Moreover, some of the confidence intervals were wide, indicating a high level of uncertainty around the estimates, and thus results should be interpreted with caution. This may be the result of the complexity of the relationships under investigation and/or due to data limitations.

Finally, people with a strong labor market attachment and stable employment are both more likely to be included, and to remain, in the SLOSH study [19]. While SLOSH is the largest continual data source on perceived job insecurity available in Sweden, the generalizability of the current study findings to the entire labor market must be done cautiously. Similarly, the transportability of the findings to other labor markets, with a different balance between employment flexibility and security, and social security net generosity, must be done with great care.

To summarize, our findings suggest that perceived job insecurity increases the risk of sickness absence for common mental disorders among employees in Sweden. It may be that persistent job insecurity, rather than changing job insecurity levels, increases the risk of SA due to CMD. However, as we previously noted, there are unresolved issues with respect to causation, confounding, and selection that warrant further attention. Still, SA due to CMD is costly for individuals, families, employers and societies as a whole. Therefore, continued study, alongside policy efforts to mitigate increasing rates of sickness absence due to mental disorders, including stress-related disorders, is of the utmost importance.

## Supplementary Material

ckaf023_Supplementary_Data

## Data Availability

Given restrictions from the ethical review board and considering that sensitive personal data are involved, it is not possible to make the data freely available. Access to the data may be provided to other researchers in line with Swedish law and after consultation with the Stockholm University legal department. Requests for data, stored at the Division of Psychobiology and Epidemiology, Department of Psychology, should be sent to registrator@su.se with reference to the study or directly to the corresponding author.
