# Supplementary material for: Exploring the link between perceived job insecurity and sickness absence for common mental disorders
Source: Eur J Public Health. 2025 Jun 10;35(4):650–6. doi: 10.1093/eurpub/ckaf023 (PMC12311365; doi:10.1093/eurpub/ckaf023)
Supplement: ckaf023_Supplementary_Data [file ckaf023_supplementary_data.docx]

**
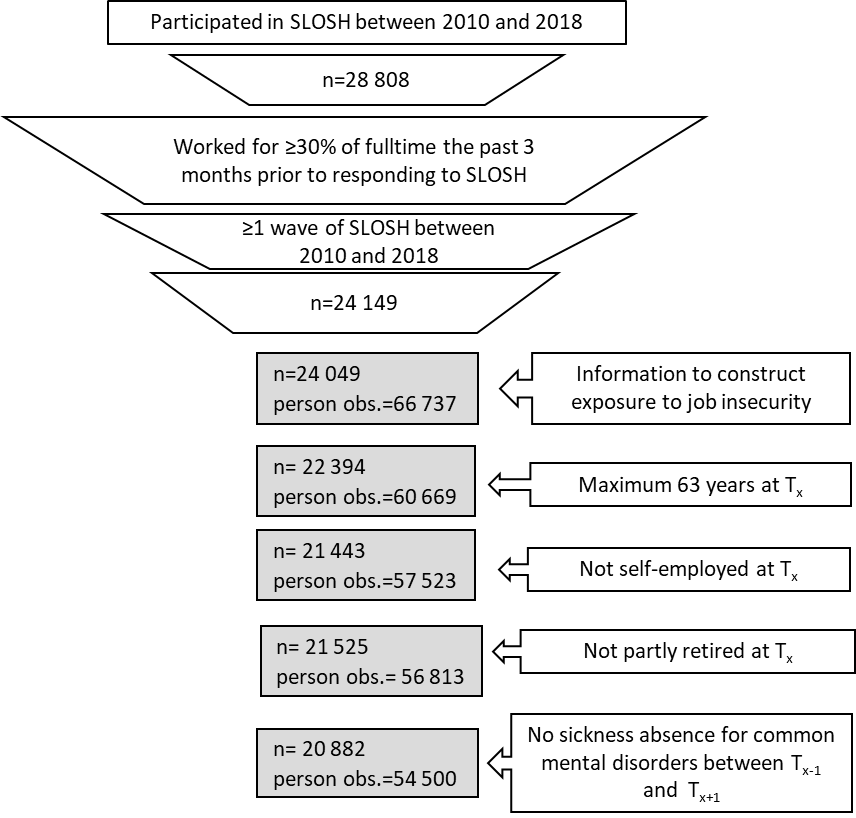
**

**Fig S1. Flow chart analytical sample step 1 analysis**

**
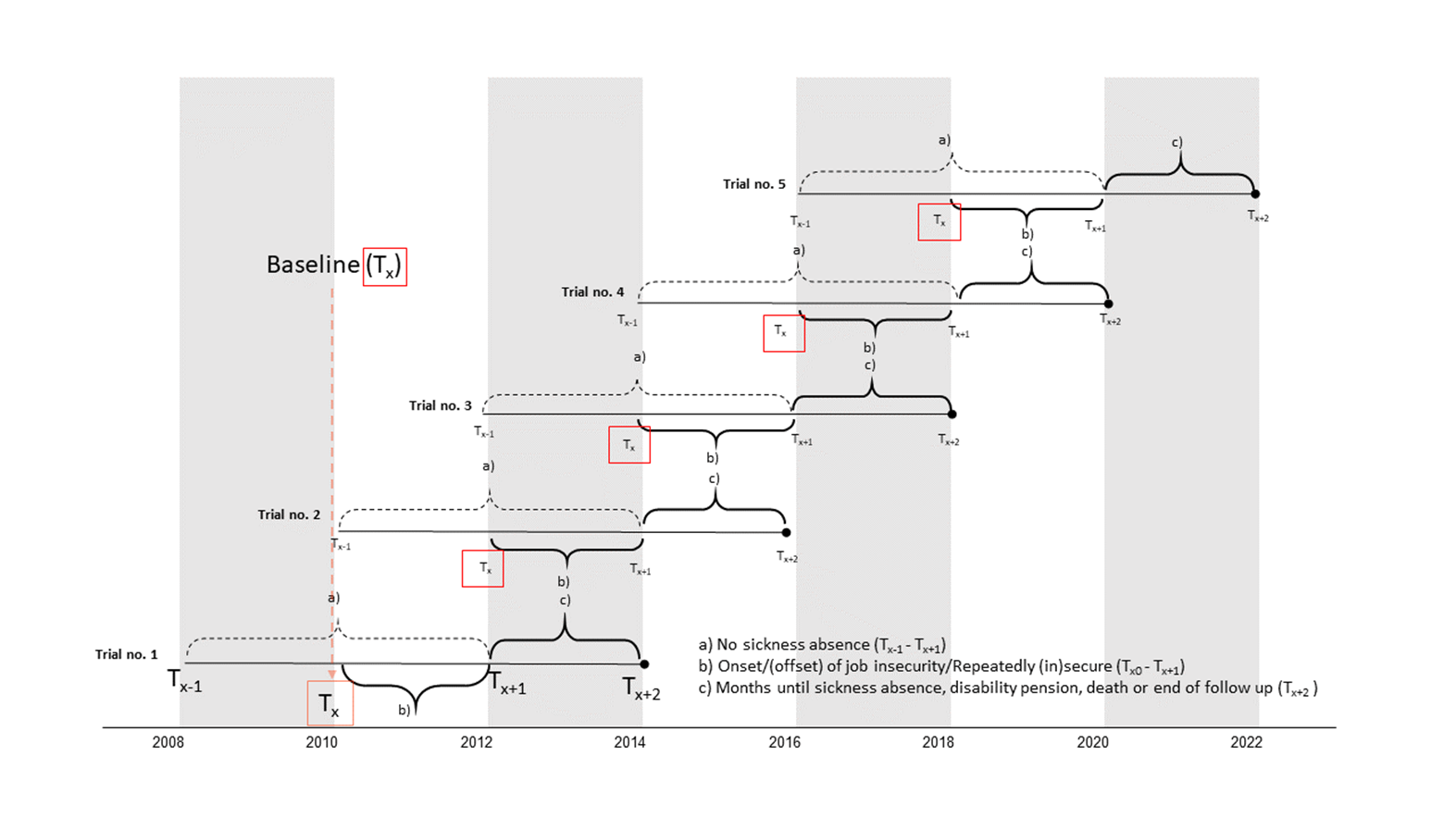
**

**Fig S2. Visualization Target Trial Emulation approach, pooling of five sequential trials**

| **Table S1. Emulated target trial protocol for an onset or offset of job insecurity and the risk of sickness absence for common mental disorders during the two successive years** | | |
| --- | --- | --- |
|  | **Target trial specification** | **Target trial emulation** |
| **Eligibility criteria** | 1) In work for at least 30% of fulltime at baseline  2) Not older than 61 years at baseline (t0)  3) Not self-employed at t0 or t1  4) Not partly retired at t0 or t1  5) No sick leave spell for psychiatric conditions during the two years prior to or at the time of the job insecurity assessment | 1 a) Working for at least 30% of fulltime the past 3 months prior to responding to SLOSH for at least two consecutive waves of data  1 b) Information needed to construct variables measuring onset and offset of job insecurity together with suitable comparison group  Criteria 2-5) Same as in the target trial specification |
| **Treatment strategy**  **q. 1** | 1) Experiencing an *onset* of job insecurity within a time period of two years, (t0 and t1)  2) Remaining free from job insecurity during the corresponding two-year period | Same as in the target trial specification |
| **Treatment strategy**  **q. 2** | 1) Experiencing an *offset* of job insecurity within a time period of two years, (t0 and t1)  2) Continuously insecure during the corresponding two-year period | Same as in the target trial specification |
| **Assignment procedures** | Individuals are randomly assigned into one of the two treatment arms for q. 1 and q. 2, respectively, at baseline and will be aware of which treatment they received | Study participants were classified according to the strategy that their data were compatible with at t0 and t1, randomization was assumed within baseline covariates |
| **Outcome** | Months until registered sick leave (SA) of ≥ 14 days due to psychiatric morbidity | Same as in the target trial specification |
| **Follow-up** | From treatment assignment and during the following two years (end of follow up), until an event (SA), disability pension, death or end of follow up, whatever comes first | During a two-year time period starting after t1 until an event (SA), disability pension, death or end of follow up (t2), whatever comes first |
| **Causal contrast** | Intention-to-treat, i.e., the effect of treatment assignment irrespective of adherence | Population average intention-to-treat effect in observational data |
| **Statistical method** | Pooled logistic regression model for standardized survival curves, risk difference, risk ratio, and counterfactual hazard ratio. | Same as in the target trial specification with sequential  emulation and adjustment for baseline covariates via inverse probability weighting for confounding adjustment and bootstrapping for valid confidence intervals |

| **Table S2.** Study participants and person observations after exclusion of ineligible respondents, by research question 1 and 2, respectively | | |
| --- | --- | --- |
| Treatment/exposure levels | ***Onset* of job insecurity comp. stably *secure*** | ***Offset* of job insecurity comp. to stably *insecure*** |
| Participated in SLOSH between 2010 and 2018 as working in 1 wave at least | n= 28 808 | |
| 1 a) Working for at least 30% of fulltime the past 3 months prior to responding to SLOSH for at least two consecutive waves of data | n=15 417 | |
| 1 b) Information needed to construct variables measuring *onset* and *offset of job insecurity* together with *suitable comparison group* | n=14 902  person obs.=34 860 | n= 1 630  person obs.=2 001 |
| 2) Not older than 61 years at baseline (t0) | n=13 578  person obs.=31 132 | n= 1543  person obs.=1 898 |
| 3) Not self-employed at t0 or t1 | n= 12 847  person obs.=29 320 | n= 1 468  person obs.=1 798 |
| 4) Not partly retired at t0 or t1 | n= 12 668  person obs.= 28 856 | n= 1 453  person obs.=1 778 |
| 5) No sick leave spell for psychiatric conditions during the two years prior to or at the time of the job insecurity assessment | n= 12 129  person obs.=26 969 | n= 1 347  person obs.=1 633 |

| **Table S3**. Baseline characteristics of the 26 969 participant-observations according to the 12 129 unique participants included from the Swedish Longitudinal Occupational Survey of Health. Characteratics are presented as part of the total number participant-observations and by level of exposure (1= Onset of job insecurity between t0 and t1 vs. 0= No job insecurity at t0 and t1). | | | | |
| --- | --- | --- | --- | --- |
|  | **Onset of job insecurity** | | **Stably secure** | |
|  | n=994 | 3.7 % | n=25 975 | 96.3% |
|  |  |  |  |  |
| **Year of Job insecurity assessment** |  |  |  |  |
| 2010-2012 | 195 | 20 | 3890 | 15 |
| 2012-2014 | 136 | 14 | 3415 | 13 |
| 2014-2016 | 227 | 23 | 7265 | 28 |
| 2016-2018 | 205 | 21 | 6236 | 24 |
| 2018-2020 | 231 | 23 | 5169 | 20 |
|  |  |  |  |  |
| Sick leave for mental disorders, within 2 years after job insecurity assessment | 42 | 4 | 801 | 3 |
| Average number of months until sick leave spell (sd) | 12.2 | (6.1) | 11.9 | (6.8) |
|  |  |  |  |  |
| Women | 540 | 54 | 15242 | 59 |
|  |  |  |  |  |
| Mean age (sd) | 48.6 | (8) | 49.4 | (8) |
|  |  |  |  |  |
| Married/cohabiting | 759 | 76 | 20909 | 81 |
|  |  |  |  |  |
| Born in Sweden | 913 | 92 | 24394 | 94 |
|  |  |  |  |  |
| Residence |  |  |  |  |
| Large city or nearby municipality | 351 | 35 | 7673 | 30 |
| Medium-sized town or nearby municipality | 371 | 37 | 10962 | 42 |
| Small town or nearby municipality | 272 | 27 | 7340 | 28 |
|  |  |  |  |  |
| Highest level of education |  |  |  |  |
| Compulsory | 327 | 33 | 7406 | 29 |
| Upper secondary | 210 | 21 | 4026 | 16 |
| University | 455 | 46 | 14531 | 56 |
|  |  |  |  |  |
| Type of Occupation |  |  |  |  |
| Manual employee | 301 | 31 | 6988 | 28 |
| Non-manual employee | 667 | 69 | 18426 | 73 |
|  |  |  |  |  |
| Sector of employment |  |  |  |  |
| Private | 646 | 68 | 12526 | 50 |
| Public | 309 | 32 | 12558 | 50 |

| **Table S4.** Baseline characteristics for the 1633 participant-observations, according to n= 1 347 unique participants included from the Swedish Longitudinal Occupational Survey of Health, by level of exposure to; 1= Offset of job insecurity between t0 and t1 vs. 0= Job insecurity at t0 and t1. | | | | |
| --- | --- | --- | --- | --- |
|  | **Job insecurity offset** | | **Stably insecure** | |
|  | n=1207 | 73.9 % | n=426 | 26.1% |
| **Year of Job insecurity assessment** |  |  |  |  |
| 2010-2012 | 248 | 21 | 103 | 24 |
| 2012-2014 | 192 | 16 | 66 | 16 |
| 2014-2016 | 361 | 30 | 131 | 31 |
| 2016-2018 | 237 | 20 | 70 | 16 |
| 2018-2020 | 169 | 14 | 56 | 13 |
|  |  |  |  |  |
| Sick leave for mental disorders, within 2 years after job insecurity assessment | 54 | 5 | 21 | 5 |
| Mean number of months until sick leave spell (sd) | 11.3 | 6.7 | 11.4 | 6.8 |
|  |  |  |  |  |
| Women | 683 | 57 | 211 | 50 |
|  |  |  |  |  |
| Mean age (sd) | 47.9 | (9) | 48.2 | (8) |
|  |  |  |  |  |
| Married/cohabiting | 893 | 74 | 339 | 80 |
|  |  |  |  |  |
| Born in Sweden | 1121 | 93 | 394 | 93 |
|  |  |  |  |  |
| Residence |  |  |  |  |
| Large city or nearby municipality | 395 | 33 | 145 | 34 |
| Medium-sized town or nearby municipality | 457 | 38 | 160 | 38 |
| Small town or nearby municipality | 355 | 29 | 121 | 28 |
|  |  |  |  |  |
| Highest level of education |  |  |  |  |
| Compulsory | 380 | 32 | 161 | 38 |
| Upper secondary | 258 | 21 | 90 | 21 |
| University | 568 | 47 | 175 | 41 |
|  |  |  |  |  |
| Type of Occupation |  |  |  |  |
| Manual employee | 394 | 34 | 140 | 34 |
| Non-manual employee | 778 | 66 | 278 | 67 |
|  |  |  |  |  |
| Sector of employment |  |  |  |  |
| Private | 696 | 61 | 289 | 70 |
| Public | 451 | 39 | 124 | 30 |

| **Table S5. Sensitivity analysis** Population average intention-to-treat effects of experiencing an *onset* or *offset* of job insecurity on sickness absence due to common mental disorders up to 2 years later, compared to remaining stably (in)*secure.*  Survival probabilities, risk differences and risk ratios adjusted for baseline confounders by inverse probability weighting, and robust 95% confidence intervals from 500 bootstrap samplings. | | | | | | |
| --- | --- | --- | --- | --- | --- | --- |
| **Allowing sickness absence for common mental disorders during two years prior to the first Job insecurity assessment** | | | | | | |
| **After 24 months of follow up** | **Secure** | | | **Onset of job insecurity** | | |
| Pers obs. 27656 (12397 unique id), SA obs=919 | est. | 95% CI | | est. | 95% CI | |
| Survival probability (95%CI) | 0.97 | 0.96 | 0.97 | 0.96 | 0.94 | 0.97 |
| Risk difference (95%CI) | Ref. |  |  | 0.01 | -0.005 | 0.03 |
| Risk ratio (95%CI) | Ref. |  |  | 1.37 | 0.91 | 1.83 |
|  |  | | |  | | |
| Pers obs. 1686 (1395 unique id), SA obs=86 | **Insecure** | | | **Offset of job insecurity** | | |
| Survival probability (95%CI) | 0.94 | 0.92 | 0.97 | 0.95 | 0.94 | 0.97 |
| Risk difference (95%CI) | Ref. |  |  | -0.01 | -0.04 | 0.02 |
| Risk ratio (95%CI) | Ref. |  |  | 0.81 | 0.36 | 1.27 |

| **Table S6. Sensitivity analysis.** Population average intention-to-treat effects of experiencing an *onset* of job insecurity on sickness absence due to common mental disorders up to 2 years later, compared to remaining stably *secure.*  Survival probabilities, risk differences and risk ratios adjusted for baseline confounders by inverse probability weighting, and robust 95% confidence intervals from 500 bootstrap samplings. | | | | | | |
| --- | --- | --- | --- | --- | --- | --- |
| **Censoring at sickness absences for other diagnoses than for common mental disorders.** | | | | | | |
|  | **Secure** | | | **Onset of job insecurity** | | |
| Pers obs. 26969 (12129 unique id), SA obs.=843 | est. | 95% CI | | est. | 95% CI | |
| **After 24 months of follow up** |  |  |  |  |  |  |
| Survival probability (95%CI) | 0.97 | 0.97 | 0.97 | 0.96 | 0.94 | 0.98 |
| Risk difference (95%CI) | Ref. |  |  | 0.01 | -0.01 | 0.03 |
| Risk ratio (95%CI) | Ref. |  |  | 1.39 | 0.78 | 2.01 |
| **Job insecurity with an increased contrast between exposed and unexposed.** | | | | | | |
|  | **Secure** | | | **Onset of job insecurity** | | |
| Pers obs. 23852 (11185 unique id), SA obs.=746 | est. | 95% CI | | est. | 95% CI | |
| **After 24 months of follow up** |  |  |  |  |  |  |
| Survival probability (95%CI) | 0.97 | 0.97 | 0.97 | 0.95 | 0.93 | 0.98 |
| Risk difference (95%CI) | Ref. |  |  | 0.01 | -0.01 | 0.04 |
| Risk ratio (95%CI) | Ref. |  |  | 1.49 | 0.75 | 2.22 |
